# Collecting Large Datasets of Unambiguous Structural Restraints for Protein Structure Determination by 4D Proton‐Detected Solid‐State NMR

**DOI:** 10.1002/cphc.202500820

**Published:** 2026-03-21

**Authors:** Veniamin Chevelkov, Sascha Lange, Adam Lange

**Affiliations:** ^1^ Research Unit Molecular Biophysics Leibniz‐Forschungsinstitut für Molekulare Pharmakologie Berlin Germany; ^2^ Institut für Biologie Humboldt‐Universität zu Berlin Berlin Germany

**Keywords:** methyl labeling, protein deuteration, protein structures, solid‐state NMR

## Abstract

Solid‐state magic‐angle spinning NMR is a powerful technique for obtaining atomic‐resolution structural information of different types of biological macromolecules. The relatively low sensitivity and limited resolution of this method have continuously stimulated methodological developments to broaden its application range. In this study, we efficiently apply proton detection, moderate sample rotation, nonuniform sampling in four‐dimensional spectra, multiplexing, deuteration, and stereospecific labeling of methyl groups to collect several hundred unambiguous long‐range distance restraints based on proton–proton magnetization transfers. These data provide a reliable description of the core domain structure of full‐length bactofilin BacA, which belongs to a newly discovered class of cytoskeletal proteins. The work demonstrates the power of combining available techniques for fast and reliable atomic‐level structural investigations that can be applied to a wide range of proteins.

## Graphical abstract

Proton detection, nonuniform sampling in 4D spectra, multiplexing, deuteration, and stereospecific labeling of methyl groups allowed for the collection of several hundred unambiguous long‐range distance restraints by solid‐state NMR for the rigid core of the bactofilin BacA.

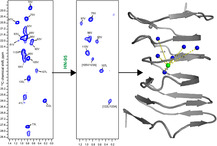

## 1 Introduction

In recent years, magic‐angle spinning (MAS) NMR spectroscopy has demonstrated tremendous progress as a technique for atomic‐level structural and dynamic characterization of various biomolecules owing to ongoing developments in instrumentation, sample preparation, and methodology [1]. Importantly, it can efficiently characterize noncrystallizable and insoluble proteins and large protein complexes that are difficult or impossible to study using X‐ray diffraction, liquid‐state NMR, or cryo‐EM techniques. The inherently low sensitivity and resolution of MAS NMR continuously demand methodological improvements to overcome these obstacles. Proton detection is a powerful approach to boost sensitivity, making use of the high gyromagnetic ratio of protons [2, 3]. These types of experiments require decreased proton density and/or high‐frequency sample rotation to attenuate strong proton–proton dipolar couplings and to obtain high‐quality spectra. Currently, probe heads that can provide sample spinning up to 160 thousand rotations per second are commercially available and open the way for collecting proton‐detected spectra.

The proton density in proteins can be reduced during bacterial expression in a D_2_O‐based medium, with deuterated sole carbon sources, and a variety of precursors with different isotope‐labeling patterns. An easy and straightforward way to achieve a low protonation level is perdeuteration with subsequent proton back‐substitution on hydrogen‐exchangeable sites in a buffer containing a H_2_O–D_2_O mixture [4, 5, 6]. This approach yields high‐resolution and sensitive spectra at MAS rates of 12 kHz and above at proper adjustment of the D_2_O concentration [6]. At a 40 kHz MAS rate and 100% reprotonation level, many proteins yield high spectral resolution, which can be substantially improved by further increasing the sample spinning up to 60 kHz [7] and even 100 kHz [8]. This effect shows that even at 40 and 60 kHz, the residual proton–proton dipolar couplings still significantly broaden proton resonances, despite the fact that only 1.7 protons per amino acid are retained from the average value of 8 protons per amino acid in the case of fully protonated proteins. At high MAS rates *ν*_r_ and uniform proton distribution, the residual dipolar line width *l*_w_ in rigid solids follows the relation [9]:

(1) $$l_{w}\propto \frac{\rho^{2}}{\nu_{r}}$$

where *ρ* is the local proton density, which defines the average internuclear distances and the related dipolar couplings. It can be seen that the MAS rate needs to increase 4 times to compensate for a double increase in proton density [10]. Centrifugal forces proportionally increase with the second power of the MAS rate, which requires a reduction in the diameter and thus volume of the rotor to maintain integrity at higher rotational rates. Therefore, the application of higher MAS rates in order to narrow proton lines reduces protein volume and might reduce overall sensitivity. The relationship between the rotor volume, maximum available spinning rate, and estimated achievable sensitivity for amide protons is presented in Table S1. These data can be used as a starting point to find a suitable combination of rotor size, spinning rate, and proton density, defined by a labeling scheme, to achieve desirable resolution and sensitivity.

Unfortunately, studies on proteins carrying protons only at labile sites provide insufficient information on side chains, which are very important for determining the overall protein structure and especially intermolecular contacts in supramolecular assemblies. Carbons can be randomly protonated by growing cells in a minimal medium containing a H_2_O/D_2_O mixture and protonated or deuterated glucose [11, 12, 13]. This scheme yields a random proton distribution over side chains with population probability dependent on the hydrogen position. At widely used MAS rates of 40–60 kHz, the protonation level should be considerably less than 100% to maintain a high resolution, which results in decreased sensitivity of individual peaks. Moreover, the intensity of the cross‐peaks based on interproton magnetization transfer is further reduced because it is proportional to the product of the proton density at the source and destination sites. Selective labeling can be used to overcome this obstacle and significantly increase the sensitivity of a specific position without a notable decrease in resolution. The success of this approach relies on the availability of suitable biosynthetic methods. Protein studies using solid‐state NMR have successfully applied selective labeling of methyl [14] and aromatic [15] groups, as well as Hα [16]. Methyl groups of different amino acids can be selectively labeled with one, two, or three protons at a high probability and without the noncontrollable protonation of other carbon positions [17, 18, 19]. This labeling scheme is attractive because of the high abundance of methyl groups, which define the hydrophobic core of proteins and intermolecular contacts in supramolecular assemblies. Currently, simultaneous labeling of all methyl groups using a single precursor is not possible; however, Leu and Val can be readily labeled together because they rely on the same α‐keto acid for biosynthesis. Valine and leucine account for approximately 20% of all residues and they are well dispersed over the primary sequence. The intraresidual dipolar couplings between the amide and methyl protons are relatively weak because of the large distance and high methyl group mobility, suggesting that the resolution would not be notably deteriorated. Experimental studies have shown high proton resolution in such systems [20]. More importantly, weak intraresidual amide–methyl interactions do not significantly quench long range proton–proton magnetization transfers induced by weak dipolar couplings—a phenomenon known as dipolar truncation [21, 22]—and do not distort the observation of important long‐range distance restraints. Several precursors and relevant protocols have been introduced over the past two decades [17] for the selective labeling of different methyl groups. A very valuable method was proposed by Boisbouvier and coworkers [23] for the stereospecific labeling of pro‐S methyl groups in valine and leucine using a 2‐acetolactate precursor. The labeling protocol offers almost 100% labeling efficiency for valine and leucine pro‐S methyl groups without detectable coexisting isotopomers and scrambling into other methyl groups. The protonation level of the pro‐S methyl group is increased by a factor of two compared to other known methods and boosts the intensity of the cross‐peaks in methyl–methyl NOESY‐type experiments by a factor of four. Eliminating the resonances of the pro‐R methyl groups also simplifies the methyl spectra without substantial loss of structure‐related information.

This methodology was employed to elucidate structure [14] and dynamics [24] by ssNMR using selective labeling of Val and Leu based on α‐ketoisovalerate protonated at a single methyl group. In this case, each Val and Leu is labeled either at the Pro‐S or Pro‐R group with 50% probability. Later, Griffin and coworkers employed a combination of precursors to introduce ILV labeling to study the structure of Influenza A M2 [25]. The precursor employed for LV labeling [23] yielded 100% labeling of only the Pro‐S methyl groups, which facilitated a significant improvement in resolution and sensitivity.

Internuclear distance information can be derived from magnetization transfers driven by dipolar couplings, which are directly defined by the internuclear distance. Protons have the strongest interspin couplings, and are thus favorable to measure. Because of the high MAS rates and low proton density, ^1^H‐^1^H interactions are significantly suppressed, and RF pulse sequences are required to recover dipolar couplings. RFDR [26, 27, 28, 29] is the most commonly used recoupling method under these experimental conditions, although DREAM [14, 30] and spin diffusion [31] in the rotating frame have been employed in several experiments as well.

Multidimensional experiments are an effective method in NMR to overcome inherently poor resolution and unambiguously access site‐specific information using correlations of nuclear resonance frequencies according to a suitably tailored experimental protocol. Deuteration and high MAS rates allow for the efficient extension of the commonly used ^13^C and ^15^N spectral dimensions by observing an additional ^1^H dimension. Under these experimental conditions, no high RF power is required to obtain long‐lived polarization [32], and 3D or even 4D experiments with long chemical evolution periods can be readily recorded to further increase the resolution, which facilitates backbone resonance assignment [33] and the collection of unambiguous long‐range distance restraints [20, 34]. Internuclear spatial proximity can be obtained by an experiment in which the frequency encodes the initial magnetization on its origin with consequent transfer through space, followed by frequency encoding on the destination nuclei. Amide–amide proton contacts can be obtained by a routine 3D H–N–(H)H experiment [26, 28, 29] that characterizes source magnetization by 2D correlations of proton and directly bound nitrogen resonance frequencies, and only a 1D spectrum describing the destination spins. In many cases, 2D HN correlations are well resolved, while 1D correlations possess severe spectral overlap that strongly limits the number of observed unambiguous restraints. To extend the available distance restraints, a variety of 3D schemes have been introduced, which combine ^1^H–^15^N or ^1^H–^13^C 2D correlations to encode magnetization on source or destination nuclei, while the conjugated dimension encodes the ^1^H, ^13^C, or ^15^N resonance frequency. By employing two complementary 3D experiments, Linser, Reif, and coworkers [28] obtained ca. 150 unambiguous ^1^H–^1^H long‐range distance restraints for a small, highly deuterated globular protein carrying amide and methyl protons. Numerous studies on larger proteins based on 3D spectroscopy have demonstrated a limited number of unambiguous long‐range restraints. Four‐dimensional spectroscopy greatly improves the resolution by employing double frequency encoding for the source and destination sites [14, 35, 36]. This strategy yields almost single‐residue resolution for many proteins. Complete sampling of all points in the indirect dimensions results in a prohibitively long measurement time on the order of weeks. However, the experimental time can be drastically reduced by nonuniform sampling (NUS) of only a small fraction of the full 4D space with subsequent spectral reconstruction according to the available algorithms [37]. NUS facilitated the assignment and structural elucidation of several proteins by ^13^C and ^1^H detected ssNMR [34, 36, 38]. In previously reported proton‐detected 4D NUS ssNMR studies, the number of distance restraints was limited. This asks for methodological improvements in these experiments to increase their efficiency and provide more structural information for better characterization of larger biomacromolecules.

In this article, we combine efficient MAS NMR and biosynthetic methods to collect several hundred unambiguous long‐range distance restraints for the core domain of the full‐length bactofilin BacA from *Caulobacter crescentus*. High resolution and sensitivity were achieved by employing proton detection at moderate MAS rates on a perdeuterated protein selectively protonated at the Val and Leu pro‐S methyl positions and 50% reprotonated at labile positions. Long‐range distance restraints were obtained based on dipolar‐driven proton–proton magnetization transfers by 4D spectroscopy employing NUS and a multiplex procedure [39, 40] for a considerable reduction of measurement time. The collected data reliably characterized the structure of the rigid core domain of the full‐length bactofilin BacA, which belongs to a recently discovered class of cytoskeletal proteins, further confirming our previously determined structure [34].

## 2 Experimental Section

### 2.1 Sample Preparation

The expression, purification, and polymerization of full‐length BacA were performed as described before [41] and is described in detail in the Supporting Information. In short, two samples with different isotope labeling schemes were used for the long‐range distance determination and resonance assignment. For long‐range distance determination, we produced a perdeuterated, uniformly ^15^N labeled protein and with stereospecifically labeled Leu‐δ2 and Val‐γ2 positions with [^13^C^1^H^2^H_2_] groups. Hereafter, this sample is referred to as the VL sample. The protein was produced in 99.9% D_2_O medium with ^15^ND_4_Cl as the sole nitrogen source, while 2‐[^13^C^1^H^2^H_2_]methyl‐4‐[^2^H_3_] acetolactate was used as a precursor for methyl labeling. The employed labeling protocol relies only on a single precursor, which simplifies sample production while providing plenty of well‐resolved and intense signals uniformly distributed across the protein. The sample was back‐protonated at the labile sites at ca. 50%. Approximately 9 mg of the protein were packed into a 1.9 mm rotor.

For assignment experiments, we produced perdeuterated, uniformly ^15^N‐ and ^13^C‐labeled full‐length BacA, with ∼ 80% reprotonation level on labile sites and ca. 10% protonation level on carbon sites, hereafter referred to as the RAP sample [11] (reduced adjoining protonation). The sample was produced by growing the bacteria in minimal medium containing ca. 10% H_2_O and 90% D_2_O^11^ for sparse proton labeling at the carbon sites. ^15^NH_4_Cl and D‐^13^C_6_‐glucose were used as sole nitrogen and carbon sources, respectively. Approximately 9 mg of protein was packed into a 1.9 mm rotor.

### 2.2 Solid‐State NMR Spectroscopy

Solid‐state NMR experiments were conducted on a standard‐bore Bruker Biospin spectrometer operating at 21.2 T external magnetic field (corresponding to a 900 MHz ^1^H resonance frequency) and equipped with a (^1^H, ^13^C, ^15^N, ^2^H) four‐channel 1.9 mm probe head. All reported experiments were based on proton detection to achieve maximum sensitivity, resulting from the high proton gyromagnetic ratio [42]. The MAS rate was set to 40 kHz, and a cooling gas flow of 1000 l/h was used to adjust the effective sample temperature to 14 ± 2°C, as measured by the temperature‐dependent water proton resonance relative to an internal DSS reference [43] (see Figure S2). This method has a relatively large error. Chemical shift referencing was achieved using the same internal DSS reference. A deuterium signal was used for the external field‐frequency lock during the measurements

The VL sample has only a few ^13^C labels and cannot be used to assign backbone and methyl group resonances, which requires an additional sample with appropriate labeling. Tugarinov and Kay [44] suggested the use of dedicated precursors to produce perdeuterated, ^13^C uniformly labeled proteins with selectively protonated methyl groups and optionally labile hydrogen positions. This strategy was recently employed in ssNMR by our lab [20]. Additionally, ultrafast MAS also allowed the assignment of methyl groups even in a fully protonated protein [45] without the need for specific labeling. For the assignment, we here employed a RAP [11] sample, which is straightforward to produce. Although the methyl protonation level is relatively low, it is still sufficient to characterize the signals in smaller systems, as demonstrated by Reif et al [46]. This approach does not require ultrafast MAS or special precursors for specific protein labeling.

Backbone chemical shift assignment was obtained using four 3D spectra recorded according to protocols presented earlier [33, 47]. Interspin magnetization transfers in (H)CANH, (H)CONH, (H)CACONH, and (H)COCANH experiments were achieved by employing only hetero‐ and homonuclear dipolar recoupling techniques, such as cross polarization (CP) [48], SPECIFIC‐CP [49], HORROR [50], and DREAM [30]. During the chemical shift evolution periods, a WALTZ‐16 [51] pulse train was applied to remove heteronuclear couplings.

The assignment of the methyl groups was obtained by the (H)CCH experiment, which correlates methyl protons and carbon to directly bound carbon nuclei (Figure S3). The experiment followed the concept earlier developed for solution‐state NMR [44] and was previously applied to proteins by solid‐state NMR to study side chains in partially and fully protonated proteins [45, 46, 52]. In the current implementation, proton–carbon and carbon–carbon magnetization transfers were achieved using CP and INEPT [53,] respectively. Dipolar and scalar proton–carbon heteronuclear couplings were suppressed by a WALTZ‐16 pulse train, and carbon–carbon scalar couplings were removed by employing constant time evolution periods. The assignment of previously reported carbon side‐chains [41] was sufficient to identify methyl protons using two known carbon frequencies. Additionally, this assignment was confirmed by two 3D (H)CHH and (H)C(HH)NH experiments utilizing a 3 ms RFDR element to induce proton–proton magnetization transfer to correlate the methyl proton and carbon or methyl carbon to amide proton or amide proton and nitrogen, respectively.

**FIGURE 1:**
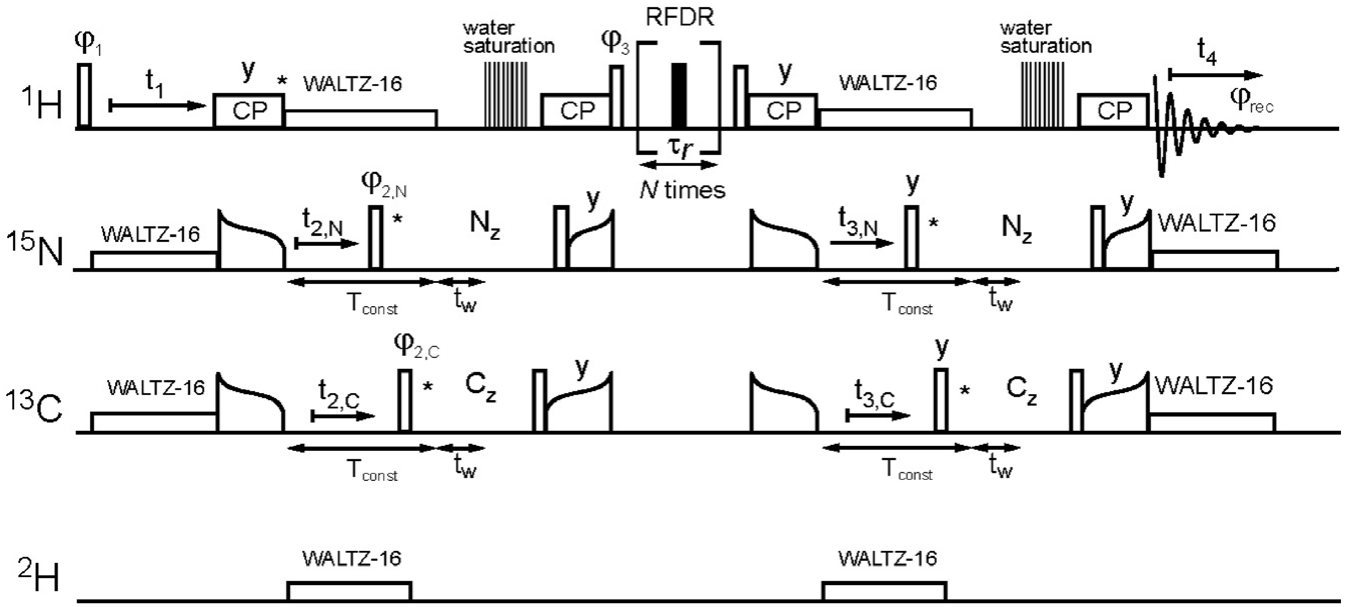
Pulse sequence for the four‐dimensional proton–proton magnetization transfer H–N/C–(HH)–N/C–H time‐shared experiment. The open and filled bars represent 90° and 180° hard pulses, respectively. Other notations representing different pulse sequence steps follow common rules. Pulses marked by asterisks were cycled to achieve quadrature detection in indirect dimensions, according to the States‐TPPI scheme. All pulses were applied along the *X*‐axis, unless stated otherwise. Phase cycling is *φ*_1_ = (*x*, *x*, –*x*, –*x*), *φ*_3_ = (*y*, −*y*), *φ*_rec_ = (*x*, *–x*, *–x*, *x*). One experiment was conducted by employing *φ*_2,N_ = *y*, *φ*_2,C_ = *y*, whereas the other experiment used *φ*_2,N_ = *y*, and *φ*_2,C_ = –*y* phase values. *T*_const_ is a constant time period to achieve equal irradiation time during each increment. Water suppression was achieved by a delay *t*_w_ and a proton saturation pulse train [54].

Long‐range distance restraints were obtained by a 4D H–N/C–(HH)–N/C–H experiment employing a finite‐pulse radio frequency‐driven recoupling (fp‐RFDR) period for the magnetization transfer between methyl and amide protons. The pulse sequence is represented in Figure 1 and it follows the “time‐shared” concept [28, 55] introduced earlier in multidimensional solution and solid‐state NMR studies. In this experiment, magnetization between spins was transferred by recoupling homo‐ and heteronuclear dipolar interactions using fp‐RFDR and CP. Finite‐pulse RFDR is a common technique for proton–proton magnetization transfer in deuterated and fully protonated samples under a broad range of MAS rates [3].

According to numerical simulations conducted with SIMPSON [56] on systems with two or three interacting protons, the rate of magnetization transfer increases almost linearly with the duration of the proton 180° pulse, as illustrated in Figure S4. This suggests that a low proton RF field of approximately 70 kHz is optimal, although a 50 kHz field might be more suitable for mixing times within 1 ms, which are also used in these types of experiments. Experimental optimizations demonstrated strong proton longitudinal polarization decay at weak proton RF field values in the range between 50 and 80 kHz. As a compromise we used in the 4D experiment 90.9 kHz proton RF field to achieve efficient mixing and minimize polarization losses. Under such conditions, only 30% of the proton signal is lost after 4.8 ms of recoupling (see Figure S5, panels B and C). To the best of our knowledge, these are the smallest magnetization losses for a proton–proton RFDR element reported so far.

A WALTZ‐16 pulse train was applied to remove relevant heteronuclear couplings during chemical shift evolution periods. The labeling scheme removes any heteronuclear and homonuclear scalar couplings between the nitrogen and carbon nuclei. To achieve high resolution, maximum indirect ^15^N and ^13^C evolution periods *t*_2,N_ and *t*_2,C_ were set to 13.3 and 14.6 ms, respectively, while evolution periods *t*_3,N_ and *t*_3,C_ were set to 12.5 and 13.7 ms, respectively. The proton maximum indirect evolution time was set to 7.3 ms. NUS was scheduled according to the Poisson‐gap distribution [37]. Four‐dimensional spectra were obtained by applying the sparse multidimensional iterative lineshape enhanced reconstruction (SMILE) protocol [57]. A single 4D experiment was recorded within approximately 5 days by sampling 23% of the data points. The experimental details and processing parameters are presented in Tables S2 and S3, respectively.

## 3 Results and Discussion

In our previous studies [34, 41], we obtained high‐quality ^13^C and ^1^H solid‐state MAS NMR spectra of BacA, which comprises 184 residues. Extensive NMR research has allowed the determination of the atomic structure of the rigid core domain (residues 39–139) of the protein by combining unambiguous restraints derived from 4D HN–HN and 2D C–C correlation spectra. Flexible N‐ and C‐terminal regions were not observed in either carbon‐ or proton‐detected NMR spectra.

It was later shown by the group of Jan Löwe that BacA assembles head‐to‐head and tail‐to‐tail [58]. This study also confirmed by X‐ray crystallography on a nonpolymerizable version of BacA the structure of the subunit earlier determined by us.

In the current study, we aimed to extend the structural characterization of full‐length BacA. The samples yielded high‐quality proton detected spectra obtained on a 900 MHz spectrometer at a 40 kHz MAS rate. Figure S6 shows the high‐resolution (H)NH and (H)CH 2D heteronuclear correlation spectra obtained for the VL sample, which was selectively protonated at the Val and Leu pro‐S methyl positions and 50% reprotonated at labile positions. The average amide and methyl proton linewidths were 80 Hz, whereas ^15^N and ^13^C linewidths were 37 and 43 Hz, respectively.

A set of three‐dimensional, dipolar‐based experiments that established intra‐ and interresidual correlations of ^15^N, ^13^Cα, ^13^CO, and ^1^H^N^ spins yielded assignments for residues S40 to F137, located in the rigid core of the protein. Signals in the flanking N‐ and C‐terminal regions were not observed in the CP‐based experiments, which is consistent with the results of our previous studies. Additionally, an INEPT‐based HN 2D correlation spectrum shows a set of signals with ^1^H chemical shifts characteristic for unfolded regions of proteins. A number of residues demonstrated signal splitting due to sample polymorphism (see Figure S7). Five out of eight Val and 13 out of 14 Leu methyl signals in the rigid core were assigned, whereas one methyl signal was not identified.

A four‐dimensional H–N/C–(HH)–N/C–H experiment [28] was employed to collect long‐range distance restraints originating from methyl and amide proton–proton magnetization transfers driven by recoupled dipolar interactions (Figure 1). The pulse sequence developed by Reif and coworkers [28] was adopted by incorporating an appropriate phase cycle on the ^13^C channel allowing for multiplexing to reduce the measurement time. The initial magnetization is double frequency encoded by the chemical shift evolution of a proton and directly bound nitrogen or carbon during the *t*_1_ and *t*_2_ evolution periods, respectively. After this step, magnetization is transferred to other protons using the fp‐RFDR technique, which is applied to recover dipolar interactions that are otherwise strongly suppressed in the sparse proton network by the MAS rate employed here. The transferred magnetization is subsequently frequency encoded by the chemical shift evolution of nitrogen or carbon and their directly bound protons during the *t*_3_ and *t*_4_ time periods, respectively. The magnetization frequencies encoded in the (*t*_1_,*t*_2_) and (*t*_3_,*t*_4_) subspaces yield 2D H‐N and H‐C correlation spectra, which are linked by the magnetization transfer between protons close together in space. High MAS rates, extensive deuteration, and sample homogeneity provided almost single‐residue resolution in the HC and HN 2D spectra (Figure S6). This, in turn, facilitates the collection of large amounts of unambiguous information about distance restraints in the 4D spectrum, although the latter has a notably lower resolution.

The time‐shared concept [59] was used for the simultaneous chemical shift evolution of the ^13^C and ^15^N nuclei during periods *t*_2_ and *t*_3_. The carbon carrier frequency was placed in the middle of the methyl region, and the bandwidth was adjusted to cover only the methyl region (∼9.7 ppm). The nitrogen carrier frequency was approximately 119 ppm and the spectral bandwidth was set to 26.4 ppm, which resulted in signal aliasing, which did not yield any resonance overlap. The spectral bandwidths of both dimensions are shown in Figure S8. Carbon and nitrogen signals in the *t*_3_ dimension can be identified according to the characteristic chemical shift of the directly bound protons, as shown in Figure S6. Recording the full proton spectral band bandwidth between 0.1 and 10.5 ppm in the indirect dimension in order to separate amide and methyl regions is extremely time consuming, which requires more efficient tailoring of the pulse sequence. In the described experiment, the proton carrier frequency was set to ∼8.1 ppm and the spectral bandwidth was adjusted to 3.4 ppm, which resulted in aliasing of the methyl region and some amide signals. The vertical dashed green lines in Figure S8 show the proton spectral width and position of the aliased methyl resonances, represented by blue peaks. The methyl region (between 0.1 and 1.2 ppm) is superimposed with the amide region between 6.8 and 7.9 ppm. By coincidence, only the Me‐41L and HN‐103K signals overlap, and only the information related to them becomes ambiguous. To remove this overlap and apply this approach in a more general case, we employed multiplexing to decode the source magnetization of amide and methyl protons into two independent polarization pathways. Two identical 4D experiments were recorded, which had only a 180° phase shift of the ^13^C 90° pulse applied along phase *φ*_2,C_ after the carbon chemical shift evolution during the *t*_2_ period. In one experiment, phases *φ*_2,N_ and *φ*_2,C_ are the same, which gives the same phase for amide and methyl signals within the subspace (*t*_1_,*t*_2_). In the second experiment, phase *φ*_2,C_ was shifted by 180° relative to *φ*_2,N_ resulting in amide and methyl signals in the opposite phase state. The sum of the two 4D spectra yields a spectrum with only an amide signal in the (*t*_1_,*t*_2_) subspace, which is then transferred by fp‐RFDR to both the amide and methyl regions. The subtraction of two 4D spectra extracts a desirable spectrum with only methyl signals in the (*t*_1_,*t*_2_) subspace, which is then transferred by fp‐RFDR to both the amide and methyl regions. Each spectrum can be readily analyzed after proper processing and calibration. Employing phase‐encoding for the initial amide and methyl polarization significantly reduces the indirect proton dimension and thus the measurement time. This pulse sequence design allowed to obtain additional proton–carbon correlations in the (*t*_1_,*t*_2_) subspace without increasing experimental time.

**FIGURE 2:**
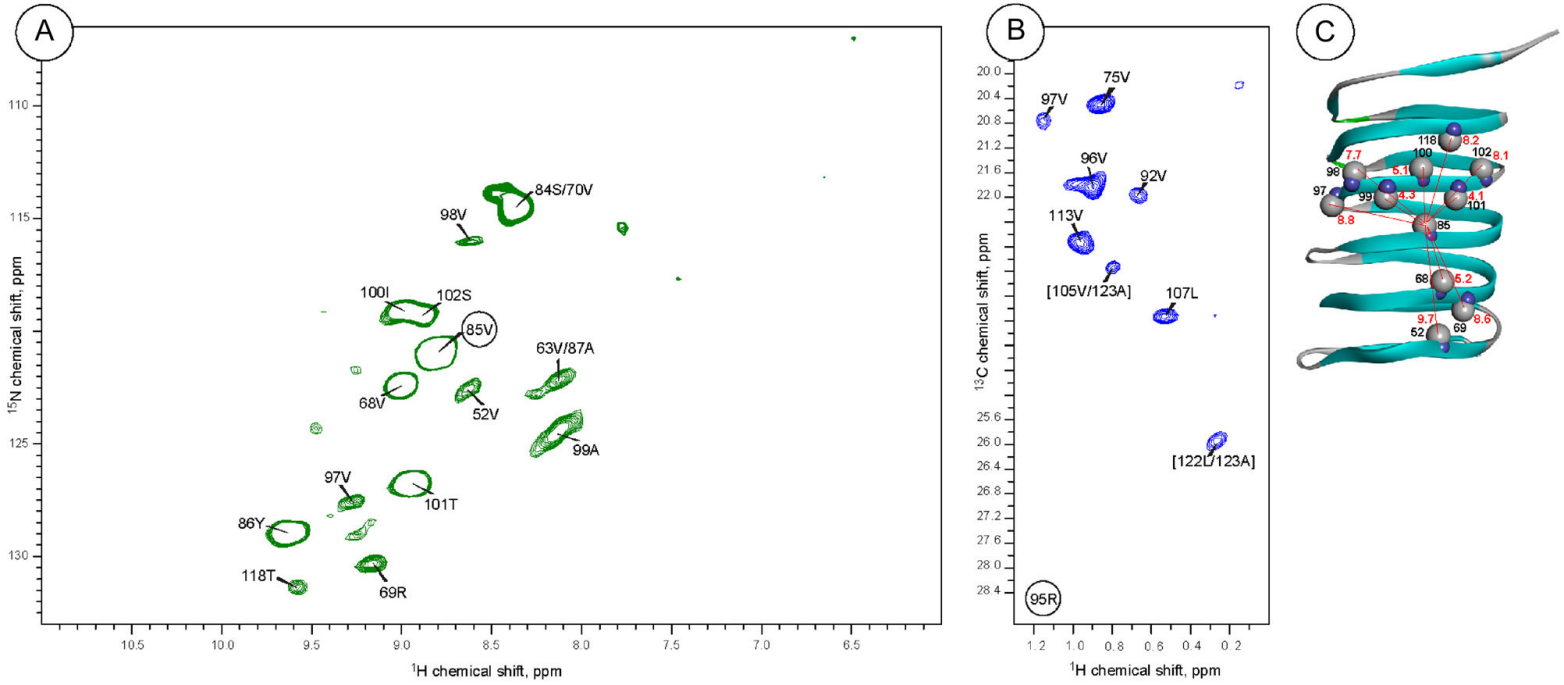
Representative 2D excerpts from 4D proton–proton magnetization transfer experiment. Panel (A) shows cross peaks arising from polarization transfer from V85‐^1^H^N^ to the amide protons, and plane (B) demonstrates cross‐peaks due to polarization transfer from R95‐^1^H^N^ to the methyl protons. The assignment possibility of unresolved resonances is separated by a slash, and the notation in the brackets (panel B) marks cross‐peaks originating from the A123‐^1^H^N^ resonance. (C) Long‐range contacts between V85‐^1^H^N^ and other amide protons displayed in the BacA structure. The residue numbers and relevant distances are shown in black and red, respectively.

The four‐dimensional spectrum yields numerous unambiguous long‐range distance restraints as a result of efficient proton–proton magnetization transfer, high sensitivity, and high resolution owing to high dimensionality. Figure 2 shows 2D excerpts that demonstrate polarization transferred from V85‐^1^H^N^ to other amide protons, and polarization transferred from R95‐^1^H^N^ to methyl protons. The spatial proton–proton contacts are defined by the intense and well‐resolved cross‐peaks represented in the 2D planes.

**FIGURE 3:**
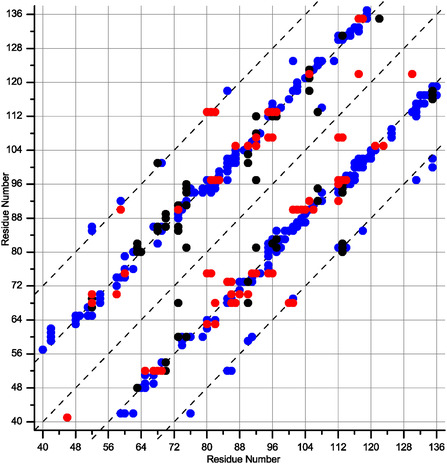
Map of unambiguous long‐range interproton contacts (between residues *i* and *j*, which follow the relation|*i–j* | > 4) as a function of the primary sequence. Blue circles represent amide–amide contacts, and black and red circles represent contacts defined by magnetization flow from methyl to amide sites and reverse. The dashed lines are diagonal and subdiagonal, defined as *y* = *x* ± 16 or *y* = *x* ± 32.

Figure 3 shows unambiguous long‐range contacts as a function of the primary protein sequence. In total, 383 unambiguous, long‐range distance restraints between residues *i* and *j* (with, |*i–j*| > 4) were identified (Table 1), while 125 contacts arose from cross peaks between methyl and amide protons, which emphasizes the power of the stereospecific methyl labeling protocol. Remarkably, the number of unambiguous contacts significantly exceeded the number of ambiguous contacts owing to the high spectral resolution. Long‐range proton–proton contacts are defined by the overall topology of the rigid BacA core domain, which is a right‐handed β‐helix with six windings and hydrophobic side chains packed in the interior. The approximate length of each winding is 16 residues, and most observed amide–amide contacts between residues *i* and *j* represent contacts between adjacent β‐strands and obey the relationship |*i–j*| ∼ 16. Figure 3 shows that most cross‐peaks are scattered around subdiagonals defined by the equation *y* = *x* ± 16 and correspond to internuclear distances within 5 Å, according to the β‐helical topology. A significant fraction of contacts between residues *i* and *j*, which considerably exceeds 5 Å, obeys the relationship |*i–j*| ∼ 32 and is created by magnetization flow between two β‐strands separated by another β‐strand between them. As an example, observed long‐range contacts between V85‐^1^H^N^ and other amide protons (see Figure 2A) were visualized in the BacA structure (PDB code: 2N3D) along the corresponding distances (Figure 2C).

**TABLE 1:** Number of long‐ and middle‐range contacts between protons from residues *i* and *j*.

Unambiguous contacts			Ambiguous contacts		
Magnetization pathway	*|* *i–j|* > 4	*|i–j|*=(3 or 4)	Magnetization pathway	*|i–j|* > 4	*|i–j|* = (3 or 4)
from $H_{i}^{N}$ to $H_{j}^{N}$	258	13	from $H_{i}^{N}$ to $H_{j}^{N}$	24	1
from $H_{i}^{\mathrm{Me}}$ to $H_{j}^{N}$	60	10	from $H_{i}^{\mathrm{Me}}$ to $H_{j}^{N}$	4	1
from $H_{i}^{N}$ to $H_{j}^{\mathrm{Me}}$	65	8	from $H_{i}^{N}$ to $H_{j}^{\mathrm{Me}}$	2	0
**∑**	**383**	**31**	**∑**	**30**	**2**

The obtained distance restraints were uniformly distributed along the primary sequence. It should be emphasized that many distances are on the order of 10 Å (i.e., twice the distance between b‐strands of 4.7 Å) and are notably longer than the typical contacts reported so far. A high degree of proton‐network dilution is extremely important for obtaining such a massive dataset. In the case of fully protonated molecules, the same amount of magnetization is distributed between significantly larger spin networks, and neighboring spins attenuate long‐range transfer owing to dipolar truncation and retain most of the magnetization. Thus, the cross‐peaks representing long‐range contacts may be reduced significantly and thus fall below the detection level. To quantify this phenomenon, we performed numerical simulations to describe the spin dynamics between two weakly coupled spins in the isolated state and in the presence of one or two additional spins with weak and strong dipolar couplings (Figure S4, S9, and S10). These simplified calculations show that neighboring contacts can reduce long‐range transfer by an order of magnitude. Several techniques have been developed to prevent the locking of magnetization within neighboring nuclei, enabling it to flow efficiently over long distances and thereby enhancing structural information. Beyond the spin dilution [28, 60] approach utilized by us in the present study, dipolar truncation can be circumvented also using spin diffusion [31] or other second‐order recoupling methods. Pulse sequences tailored for chemical shift selective recoupling [61, 62] restrict the number of interacting nuclei and localize the magnetization exchange within a limited spin space, significantly enhancing the pairwise signal transfer. In addition, magnetization dynamics over long ranges during RFDR mixing can be significantly corrected by accounting for multipin interactions, which allows for more reliable and precise quantification of long‐range contacts [63].

## 4 Conclusion

In this work, we combined several approaches that allowed us to collect several hundred unambiguous long‐range distance restraints to accurately describe the rigid core domain of the full‐length bactofilin BacA. Proton detection on deuterated samples at moderate MAS rates yielded high resolution and sensitivity in three‐ and four‐dimensional spectra. Selective protonation of the Val and Leu pro‐S methyl positions and reprotonation on labile positions at 50% provided a sufficient number of reporters covering all parts of the protein. Simultaneously, a low proton density allowed long‐range magnetization transfer driven by RFDR recoupling. Four‐dimensional spectroscopy was required to facilitate the almost single‐residue resolution necessary for the unambiguous characterization of distance restraints. NUS and multiplexing significantly reduced the measurement time. We believe that the quality of the reported data will stimulate a broader application of the presented combination of previously developed techniques for studying large and complex biomolecules using solid‐state MAS NMR.

## Supporting Information

Additional supporting information can be found online in the Supporting Information section. **Supporting** **Figure S1.** Estimated sensitivity of proton‐detected spectra as a function of rotor size. Based on the values listed in Table S1. **Supporting** **Figure S2.** Proton spectrum of the BacA protein obtained using a direct excitation pulse. DSS and water peaks are shown in insets. **Supporting** **Figure S3.** Panels A) and C) represent pulse sequences for 3‐dimensional (H)CCH experiments employed for the assignment of methyl groups. The open and filled bars represent 90° and 180° hard pulses, respectively. The corresponding coherence transfer schemes are provided at the bottom of each pulse sequence. All pulses were applied along the *X*‐axis, unless stated otherwise. The phase cycle is *φj*_1 _= (*y*,‐*y*), *φj*_2_=(*y*, *y*, ‐*y*, ‐*y*), *φj*_3_=4×(‐*y*), 4×(*y*), *φj*_4_=2×(*x*), 2×(‐*x*), *φj*_5_=8×(*x*), 8×(‐*x*), *φj*_6_=4×(*x*), 4×(‐*x*) and *φj*_rec_=(x, ‐x, ‐x, x, ‐x, x, x, ‐x, ‐x, x, x, ‐x, x, ‐x, ‐x, x). C^m^ and C^b^ denote the methyl carbon and the carbon directly bound to the methyl carbon, respectively. The delay *τt*_a_ is defined by the ^13^C‐^13^C J coupling as *τt*_a_ =1/(4J_CC_). Water suppression was achieved by delay *τt*_w_ and a proton saturation pulse train. In the experiment in panel C) all ^13^C‐^13^C J‐couplings were removed for all the observed carbons. The magnetization flow was the same for both experiments and is depicted in panel B). **Supporting** **Figure S4.** Simulated proton‐proton magnetization transfer dynamics under fp‐RFDR‐16 recoupling in a three‐proton system at different RF field strengths. The “spin system” section of the SIMPSON input file is provided below. The initial magnetization of the source spin was 1, whereas spin 2 was observed. **Supporting** **Figure S5.** Comparison of the first 1D from the 2D (H)‐N/C‐H (A) and 4D H‐N/C‐(HH)‐N/C‐H experiments with 0 and 4.8 ms of proton‐proton mixing in (B) and (C), respectively. The relative integral intensities of the amide and methyl regions are indicated next to the relevant peak. The spectrum in A) was recorded using four scans, and the spectra in B) and C) were recorded using eight scans. The spectrum in (A) was scaled up 2 times to account for the different numbers of scans. A spectral comparison showed that the CP transfer efficiency was approximately 78% and the magnetization losses during the RFDR period were 20–30%. **Supporting** **Figure S6.** Two‐dimensional proton‐detected (H)NH and (H)CH correlation spectra of 50% back‐protonated [U‐^2^H,^15^N,^13^C‐Me‐pro‐S‐VL] BacA. Cross‐polarization was employed for the inter‐spin magnetization transfer. Spectra were obtained on a 900 MHz spectrometer at an MAS rate of 40 kHz. The perdeuterated protein was back protonated at amide sites at a level of 50%. The prime and double prime signs refer to the signals from other polymorphs. Maximum acquisition times in the ^15^N and ^13^C dimensions were 40 ms and 29.3 ms, respectively. Prior to Fourier transformation, the data in the proton dimension were truncated to 16 ms and apodised with a 47°‐shifted squared sine bell window function. In the carbon and nitrogen dimension, 2 Hz of Lorentzian broadening was applied prior to apodisation. The average nitrogen and carbon linewidths measured without apodisation were 37 ± 4 Hz and 43 ±6 Hz, respectively. The spectra in panels A and B were recorded using four and eight scans, respectively. The inter‐scan delay was set to 2.5 s for both spectra. **Supporting** **Figure S7.** Schematic representation of signal splitting of backbone amide signals. Each circle represents a single signal. Residues are schematically distributed in six vertically aligned windings according to the protein topology. **Supporting** **Figure S8.** Representation of signal folding in indirect proton (t1) and nitrogen/carbon (t2) dimensions. The amide and methyl resonances are shown in green and blue, respectively. The experimental frequency bandwidth was defined by the time increments in the indirect dimensions. The green dashed lines represent the spectral bandwidths of the amide region and the blue dashed lines represent the aliased methyl regions. **Supporting** **Figure S9.** A) Simulated proton‐proton magnetization transfer dynamics under fp‐RFDR‐16 recoupling in a system of two or three interacting protons. The proton RF field strength is 90.9 kHz and the initial magnetizations of I_1_, I_2_, and I_3_ are 1, 0, and 0, respectively. Table S4 summarizes the inter‐proton couplings employed in the calculations. **Supporting** **Figure S10.** Simulated proton‐proton magnetization transfer dynamics under fp‐RFDR‐16 recoupling in a system of two or four interacting protons. The proton RF field strength was 90.9 kHz and the initial magnetization of I_1_ was 1, while the other nuclei had 0 magnetization and I_2z_ was observed. The black curve shows the I_2z_ magnetization dynamics in the system of I_1_ and I_2_ nuclei, whereas the red curve represents the magnetization in a 4‐spin system. Considered proton‐proton dipolar couplings are represented by dashed lines along the distances in the bottom figures. The proton systems in the bottom panel are color‐coded according to the curves in the main panel. The “spin system” section of the SIMPSON input file is described below. The spin system in red color represents a simplified hydrogen network in a fully protonated system consisting of two adjacent amides and two Ha protons. **Supporting** **Table S1.** Estimated sensitivity of amide groups in proton‐detected experiments for different types of MAS rotors manufactured by Bruker. All the specifications marked with ^(1)^ were provided by Bruker. **Supporting** **Table S2.** Experimental parameters of 4D experiments. **Supporting** **Table S3.** 4D processing parameters. **Supporting** **Table S4.** The active proton‐proton couplings used for the simulations that are presented in Figure S9. The right column exemplifies the “spin system” section of the SIMPSON input file for the curve “E.” The other simulations used the same parameters. The proton 180° pulse duration was set to 5.5 ms, to match the experimental conditions. 

## Funding

This study was supported by Leibniz‐Gemeinschaft (K305/2020), Deutsche Forschungsgemeinschaft (EXC 2008/1 (UniSysCat)‐−390540038).

## Conflicts of Interest

The Authors declare no conflicts of interest.

## Supporting information

Supplementary Material

## Data Availability

The data that support the findings of this study are available from the corresponding author upon reasonable request.
