# Supplementary material for: Collecting Large Datasets of Unambiguous Structural Restraints for Protein Structure Determination by 4D Proton‐Detected Solid‐State NMR
Source: Chemphyschem. 2026 Mar 21;27(6):e202500820. doi: 10.1002/cphc.202500820 (PMC13005908; doi:10.1002/cphc.202500820)
Supplement: Supplementary file 1 — Supplementary Material [file CPHC-27-e202500820-s001.pdf]

Supporting Information for

Collecting Large Data Sets of Unambiguous Structural Restraints for Protein Structure Determination by 4D Proton-Detected Solid-State NMR

Veniamin Chevelkov[a], Sascha Lange[a], and Adam Lange*[a,b]

[a] Research Unit Molecular Biophysics, Leibniz-Forschungsinstitut für Molekulare Pharmakologie, 13125 Berlin, Germany.
[b] Institut für Biologie, Humboldt-Universität zu Berlin, 10115 Berlin, Germany.
*E-Mail: alange@fmp-berlin.de

Table S1 reports the key physical characteristics of the MAS rotors manufactured by Bruker and the evaluation of the relative amide proton sensitivity in proton-detected experiments. Other companies, such as Phoenix NMR and Jeol Resonance, along with scientific groups (i.e. Samoson's lab), develop probe heads with rotor specifications that differ from Bruker's standards. However, the differences in rotor diameter, sample volume, and maximum spinning rate are limited, as they are largely determined by the physical properties of the zirconium dioxide used in the rotor fabrication.

The presented gross estimations consider a system as rigid solid (Eq. 1) which implies that this evaluation cannot be applied to highly mobile moieties such as methyl groups. Moreover, numerous hardly controllable relevant parameters, such as protein motion, which scales down dipolar interactions, non-uniform proton density, and possible influence of additional dipolar couplings with water molecules and hydrogen exchange are not controllable, but still might affect observations. The different dynamics of different proton moieties and dependence on the sample type yield different T_1 and T_2 values, which implies that comparisons should be made between proteins and protons of similar types. In addition, doping a sample with a paramagnetic agent[1] at a high concentration would practically remove the sensitivity dependence on T_1 because the repetition delay would be essentially the same for all protonation levels and be defined by hardware safety. For comparison, we considered that all the spectra have virtually the same contribution to the linewidth from the residual homonuclear dipolar couplings. This means that at lower MAS rates, the proton density should be lower, resulting in smaller proton concentration and a longer longitudinal relaxation rate. Both factors decrease the sensitivity per volume unit, whereas the overall sensitivity can be increased by increasing the sample volume in a rotor with a larger volume.

To maintain the same residual dipolar line broadening at all MAS rates, the relative proton density was derived using Eq. 1 ($l_w \propto \frac{\rho^2}{\nu_r}$). The longitudinal relaxation time defines the repetition delay and consequently influences the overall sensitivity per time unit. The longitudinal relaxation time of amide protons as a function of their density was evaluated from experimental studies by Akbey *et al.* [2]. Protons of another type might have different relaxation properties due to different mobilities, which would require other data for evaluation. Considering that the coil sensitivity is inversely proportional to its diameter, the relative sensitivity of the proton peaks in the 2D (H)NH or (H)CH spectra can be estimated as follows:

$$S/N_{2D} \propto \frac{\rho \cdot V_s}{l_w \cdot d_{coil}} \cdot \frac{1}{\sqrt{T_{1,R}}} \propto \frac{\nu_{rMAX} \cdot V_s}{\rho \cdot d_{coil}} \cdot \frac{1}{T_{1,R}} \propto \frac{\sqrt{\nu_{rMAX}} \cdot V_s}{d_{coil}} \cdot \frac{1}{\sqrt{T_{1,R}}}$$

The relative sensitivity of cross-peaks in experiments based on inter-proton magnetization transfer depends on the density in the second power, and for two isolated spins can be expressed as follows:

$$S/N_{MIX} \propto \frac{\rho^2 \cdot V_s}{l_w \cdot d_{coil}} \cdot \frac{1}{\sqrt{T_{1,R}}} \propto \frac{\nu_{rMAX} \cdot V_s}{d_{coil}} \cdot \frac{1}{\sqrt{T_{1,R}}}$$

Symbol	Meaning					
d_o[1], mm	Outer rotor diameter	0.7	1.3	1.9	2.5	3.2
ν_{rMAX}[1], kHz	Maximum spinning frequency	111	67.0	42.0	35.0	24.0
d_{coil}, mm	RF coil diameter (estimated)	1	1.7	2.4	3.0	3.7
S/N_{coil}	Relative coil sensitivity	1	0.59	0.42	0.33	0.27
V_s[1], μL	Sample volume	0.59	3.0	13.1	13.6	46.7
ρ_R	Relative proton density	1	0.78	0.62	0.56	0.46
$T_{1,R}$	Relative experimental longitudinal relaxation time	1	1.21	1.44	1.55	1.78
$S/N_{2D,R}$	Relative sensitivity in a 2D HETCOR spectrum	1	2.11	4.74	3.47	7.45
$S/N_{MIX,R}$	Relative sensitivity of proton-proton cross-peaks in NOESY-type experiments	1	1.64	2.91	1.95	3.46

Table S1. Estimated sensitivity of amide groups in proton-detected experiments for different types of MAS rotors manufactured by Bruker. All the specifications marked with [1] were provided by Bruker.

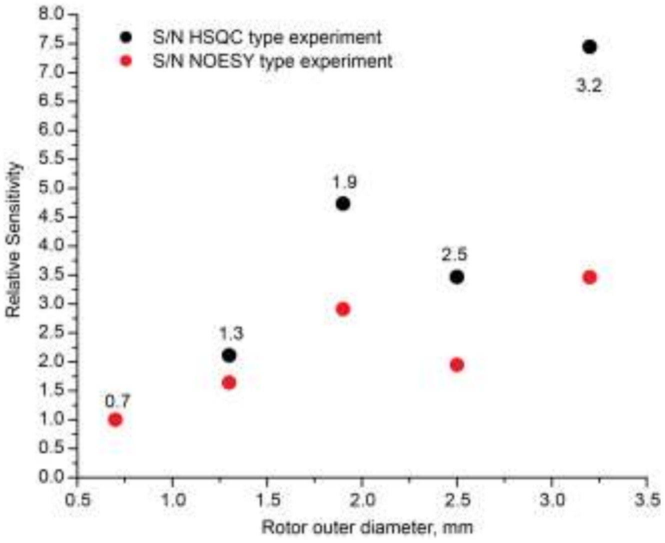

Figure S1. Estimated sensitivity of proton-detected spectra as a function of rotor size. Based on the values listed in Table S1.

Sample Preparation.

For assignment experiments, we produced perdeuterated, uniformly ^{15}N- and ^{15}C-labeled full-length BacA with ~80% reprotonation at labile sites and ~10% protonation at carbon-bound positions, hereafter referred to as the RAP sample.

A pET21a vector encoding full-length BacA was transformed into *E. coli* BL21(DE3)/pLysS (Invitrogen). A single colony was inoculated into 2 ml pre-warmed (37 °C) M9 medium containing 90% D_2O. After 4 h incubation at 37 °C and 150 rpm, the culture was diluted 1:1 with pre-warmed M9 (90% D_2O) and grown for an additional 2 h. The entire preculture was used to inoculate 50 ml M9 (90% D_2O) and incubated overnight at 30 °C (150 rpm). Subsequently, the overnight culture was diluted into 500 ml M9 (90% D_2O) to yield the final expression culture. $^{15}NH_4Cl$ and D-[U-$^{13}C_6$,1,2,3,4,5,6,6-d_7]glucose served as the sole nitrogen and carbon sources.

After growth at 37 °C (150 rpm), the temperature was reduced to 25 °C and protein expression was induced with 0.5 mM IPTG, followed by a further decrease to 20 °C. Cells were harvested after ~16 h.

Cell pellets were resuspended in lysis buffer (6 M guanidinium hydrochloride, 50 mM NaH_2PO_4, 10 mM Tris-HCl, 20 mM imidazole, 1 mM β-mercaptoethanol; pH 8.0) and lysed by sonication. After centrifugation (30 min, 20,000 rcf), the supernatant was applied to a 5 ml His-Prep HP column on an Äkta Pure 25 FPLC system. The column was washed with five column volumes of wash buffer (8 M urea, 50 mM NaH_2PO_4, 10 mM Tris-HCl, 20 mM imidazole, 1 mM β-mercaptoethanol; pH 6.4), and BacA was eluted with five column volumes

of elution buffer (8 M urea, 50 mM NaH_2PO_4, 10 mM Tris-HCl, 500 mM imidazole, 1 mM β-mercaptoethanol; pH 4.5).

The denatured protein was further purified by size-exclusion chromatography on a 26/60 Sephacryl S-300 HR column equilibrated in 8 M urea, 50 mM HEPES, 50 mM NaCl, 5 mM $MgCl_2$, 0.1 mM EDTA (pH 7.2). The protein solution was dialyzed against 5 L polymerization buffer (50 mM HEPES, 50 mM NaCl, 5 mM $MgCl_2$, 10% glycerol, 0.1 mM EDTA; pH 7.2) for ≥2 days with four buffer exchanges.

Renatured BacA was pelleted by ultracentrifugation (540,000 rcf, 8 h), resuspended in 20 mM Tris-HCl (pH 7.5, 80% H_2O/20% D_2O), and incubated for one day at 4 °C with agitation to allow for back-exchange of labile protons. A second ultracentrifugation step yielded the final polymerized protein. Approximately 9 mg were packed into a 1.9 mm MAS rotor.

For long-range distance measurements, we prepared a perdeuterated, uniformly ^{15}N-labeled sample with stereospecific ^{13}C–$^{1}H^{2}H_2$ methyl labeling at the Leu-δ_2 and Val-γ_2 positions, hereafter referred to as the VL sample.

A single transformant was grown in 5 ml of a pre-warmed 1:1 mixture of 99.9% D_2O-based M9 medium and LB (protonated). After 4 h at 37 °C (150 rpm), the culture was diluted with 5 ml M9 (99.9% D_2O) and incubated until reaching an OD_{600} of ~0.6. This preculture was used to inoculate 60 ml M9 (99.9% D_2O), followed by overnight incubation (≥16 h) at 37 °C and 150 rpm. Cells were pelleted (20 min, 3500 rcf, 37 °C) and resuspended in 500 ml M9 (99.9% D_2O) containing $^{15}NH_4Cl$ as the sole nitrogen source and D-[U-$^{12}C_6$,1,2,3,4,5,6,6-d_7]glucose as the carbon source.

Upon reaching OD_{600} ≈ 0.6, 2-[^{13}C,D_2]methyl-4-(D_3)-acetolactate (DLAM-LVProS; BioNMR) was added according to the manufacturer's instructions. One hour later, expression was induced with 0.5 mM IPTG and the temperature reduced to 20 °C. After ~16 h expression, cells were harvested.

Purification followed the same protocol as for the RAP sample. Labile protons were back-exchanged in 50% H_2O/50% D_2O. Approximately 9 mg of the final VL-labeled BacA were packed into a 1.9 mm MAS rotor.

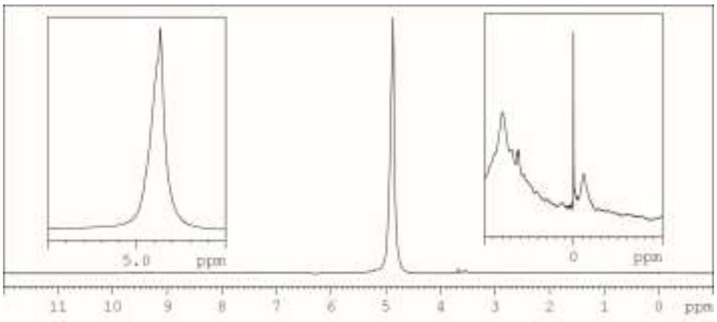

Figure S2. Proton spectrum of the BacA protein obtained using a direct excitation pulse. DSS and water peak are shown in insets.

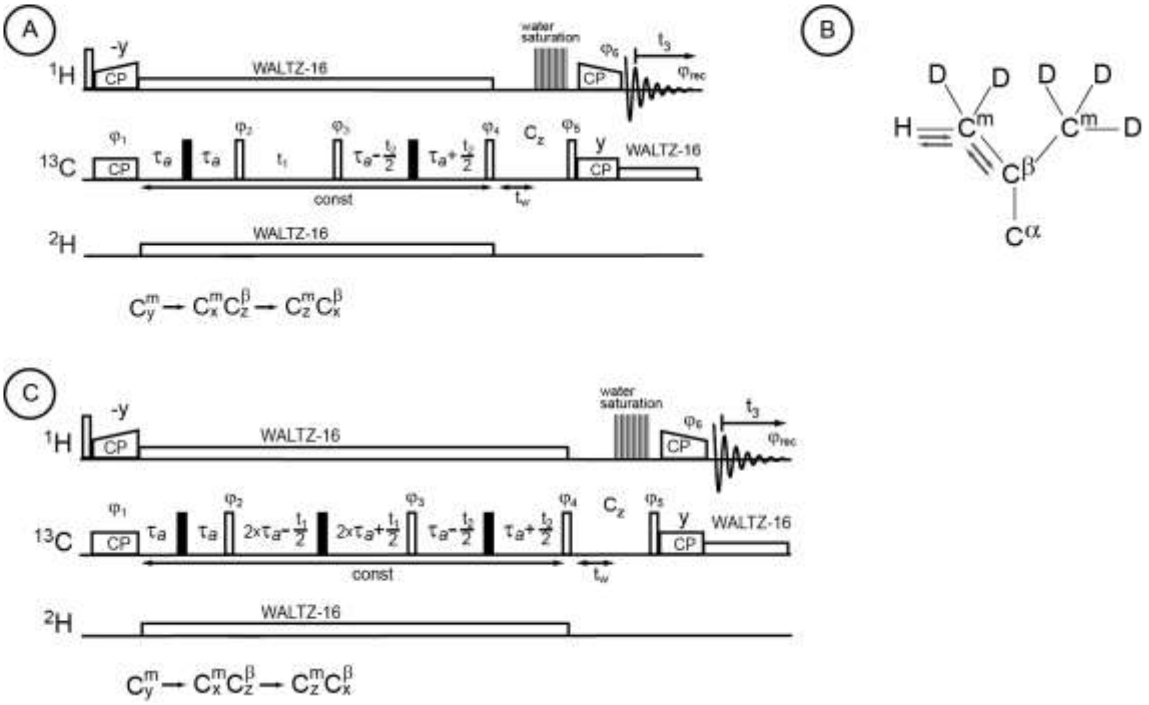

Figure S3. Panels A) and C) represent pulse sequences for 3-dimensional (H)CCH experiments employed for the assignment of methyl groups. The open and filled bars represent 90° and 180° hard pulses, respectively. The corresponding coherence transfer schemes are provided at the bottom of each pulse sequence. All pulses were applied along the X-axis, unless stated otherwise. The phase cycle is φ_1=(y,-y), φ_2=(y,y,-y,-y), φ_3=4×(-y), 4×(y), φ_4=2×(x), 2×(-x), φ_5=8×(x), 8×(-x), φ_6=4×(x), 4×(-x) and φ_{rec}=(x, -x, -x, x, -x, x, x, -x , -x, x, x, -x, x, -x, -x, x). C^m and C^β denote the methyl carbon and the carbon directly bound to the methyl carbon, respectively. The delay τ_a is defined by the ^{13}C-^{13}C J coupling as τ_a =1/(4J_{CC}). Water suppression was achieved by delay τ_w and a proton saturation pulse train. In the experiment in panel C) all ^{13}C-^{13}C J-couplings were removed for all the observed carbons. The magnetization flow was the same for both experiments and is depicted in panel B).

Figure S3 shows the pulse sequences employed to assign methyl groups. The experiments correlate the methyl protons and carbon to the directly bound carbon nuclei.

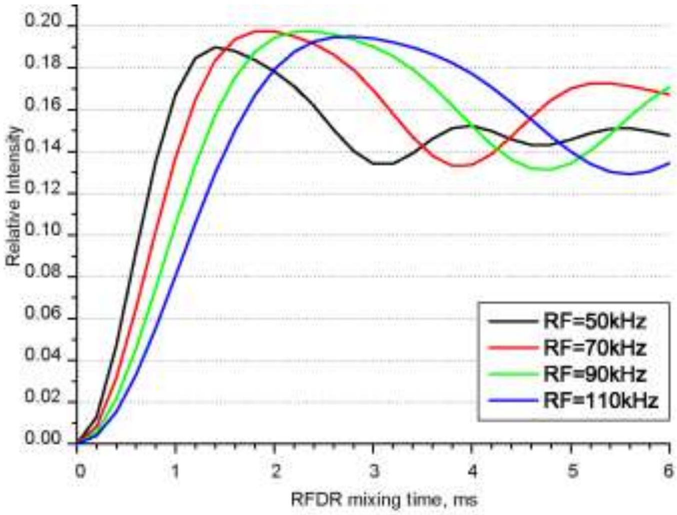

Figure S4. Simulated proton-proton magnetization transfer dynamics under fp-RFDR-16 recoupling in a three-proton system at different RF field strengths. The "spin system" section of the SIMPSON input file is provided below. The initial magnetization of the source spin was 1, whereas spin 2 was observed.

```
spinsys {
   dipole 1 2 -1743. 0 0 0
   dipole 1 3 -1743. 120 0 0
   dipole 2 3 -1743. 150 0 0
   shift 1 -4.1p 7.7p   0.65  17  84  12
   shift 2 -3.8p 7.7p 0.65  77 54 22
   shift 3 4.0p 7.7p 0.65   20 45 56
  }
```

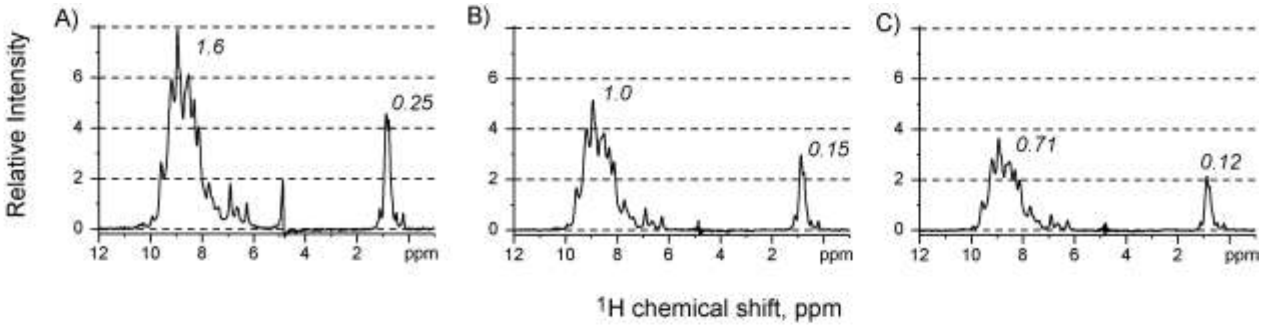

Figure S5. Comparison of the first 1D from the 2D (H)-N/C-H (A) and 4D H-N/C-(HH)-N/C-H experiments with 0 and 4.8 ms of proton-proton mixing in (B) and (C), respectively. The relative integral intensities of the amide and methyl regions are indicated next to the relevant peak. The spectrum in A) was recorded using four scans, and the spectra in B) and C) were recorded using eight scans. The spectrum in (A) was scaled up 2 times to account for the different numbers of scans. A spectral comparison showed that the CP transfer efficiency was approximately 78% and the magnetization losses during the RFDR period were 20-30%.

During 4-dimensional experiments, typical RF fields for high-power pulses were 83 kHz, 31.3 kHz, and 48.1 kHz for proton, nitrogen, and carbon, respectively. During ^{1}H-^{1}H RFDR mixing, proton pulses were cycled according to the XY-16 scheme. The proton pulse length was 5.5 ms, while the RF strength was set to 90.9 kHz. During the chemical shift evolution period, WALTZ-16 was applied to remove heteronuclear couplings. Radiofrequency field strengths were 7.4, 4.0, 4.4, and 0.9 kHz for ^{1}H, ^{13}C, ^{15}N, and ^{2}H channels, respectively, while durations of 90° pulses were 60, 70, 50, and 360 μs for ^{1}H, ^{13}C, ^{15}N, and ^{2}H channels, respectively. Other experimental parameters are listed in Table S2:

		^{1}H	^{15}N	^{13}C	
t_1 evolution					
	Sweep width, kHz/ppm	3014/3.4			
	Carrier position, ppm	8.12			
	Increment, μs	331.7			
	Number of increments	22			
	evolution time, ms	7.3			
1st CP					
	Average RF field strength, kHz	69	29	29	
	Duration, ms	2.8	2.8	2.8	
	Shape Amplitude/Angle,°	rectangular	tangential, 100-60/75	tangential, 100-60/75	
t_2 evolution					
	Sweep width, kHz/ppm		2404/26.4	2185/9.66	
	Carrier position, ppm		119.3	24	
	Increment, μs		416	457.6	
	Number of increments		32	32	
	evolution time, ms		13.3	14.6	
2nd CP					
	Average RF field strength, kHz	69	29	29	
	Duration, ms	1.7	1.5	1.7	
	Shape Amplitude/Angle,°	rectangular	tangential, 60-100/75	tangential, 60-100/75	
^{1}H-^{1}H RFDR					
	180° pulse length, μs	5.5			
	Mixing time, ms	4.8			
3d CP					
	Average RF field strength, kHz	69	29	29	
	Duration, ms	2.8	2.8	2.8	
	Shape Amplitude/Angle,°	rectangular	tangential, 100-60/75	tangential, 100-60/75	
t_3 evolution					
	Sweep width, kHz/ppm		2404/26.4	2185/9.66	
	Carrier position, ppm		119.3	24	
	Increment, μs		416	457.6	
	Number of increments		30	30	
	evolution time, ms		12.5	13.7	
4th CP					
	Average RF field strength, kHz	69	29	29	
	Duration, ms	1.7	1.5	1.7	
	Shape Amplitude/Angle,°	rectangular	tangential, 60-100/75	tangential, 60-100/75	
Decoupling					
	scheme	WALTZ-16	WALTZ-16	WALTZ-16	
	Basic pulse (90°), μs	40	50	70	
	RF strength, kHz	7.4	4.42	4.0	

WALTZ-16 decoupling applied on ^{2}H channel employed 300 μs for the basic 90° pulse.

23% sampling, number of scans is 4, recycle delay is 2.65 s

The total measurement time of a single spectrum is approximately 5 days and 2 h. The effective sample temperature was set to ca. 14 °C.

Table S2. Experimental parameters of 4D experiments.

The parameters for WALTZ-16 decoupling applied to the ^{15}N and ^{13}C channels during ^{1}H evolution were optimized by monitoring the ^{1}H amide and ^{1}H methyl 1D bulk signals, respectively. A grid search was performed over a pulse duration of 20–120 μs and RF strength of 0–10 kHz. No interference between these simultaneously applied decouplings was observed, which can be explained by the long distance between ^{13}N and ^{13}C, as the latter is present only at methyl positions. WALTZ-16 applied to the ^{2}H channel was optimized for the directly detected ^{13}C bulk signal. An RF field was scanned at low output power levels for pulse durations of 200 and 300 μs. WALTZ-16 applied to the ^{1}H channel was optimized for the maximum ^{1}H 1D bulk signal obtained after a ^{15}N spin-echo period of 24 ms in the 1D version of the (H)NH experiment. A grid search was performed for pulses in the range of 20–80 μs and RF strengths from 0 kHz to 12 kHz. No interference between decoupling applied simultaneously on the ^{2}H and ^{1}H channels was observed owing to the RF field strength difference of approximately nine times.

Extensive two parameter optimization allows the safe avoidance of the recoupling condition, although the optimization routine can be accelerated using analyses of the WALTZ-16 pulse train[3], which found that recoupling conditions occur at a 90° pulse duration of 25 μs and 62,5 μs in the case of a 40 kHz MAS rate.

	^{1}H, direct (t_4)	^{15}N, ($t_{3,N}$)	^{13}C, ($t_{3,C}$)	^{1}H, indirect (t_1)	^{15}N, ($t_{2,N}$)	^{13}C, ($t_{2,N}$)
Effective acquisition time, ms	12.8	12.48	13.73	7.3	13.3	14.63
Sweep width, Hz		2403.8	2185.3	3014.44	2403.8	2185.3
Sweep width, ppm		26.36	9.66	3.35	26.36	9.66
Number of points		128	128	64	128	128
Digital resolution, Hz/(per point)	24.4	18.8	17.1	47.1	18.8	17.1
Shift of squared sine bell window function /°	56	72	72	72	72	72

Table S3. 4D processing parameters.

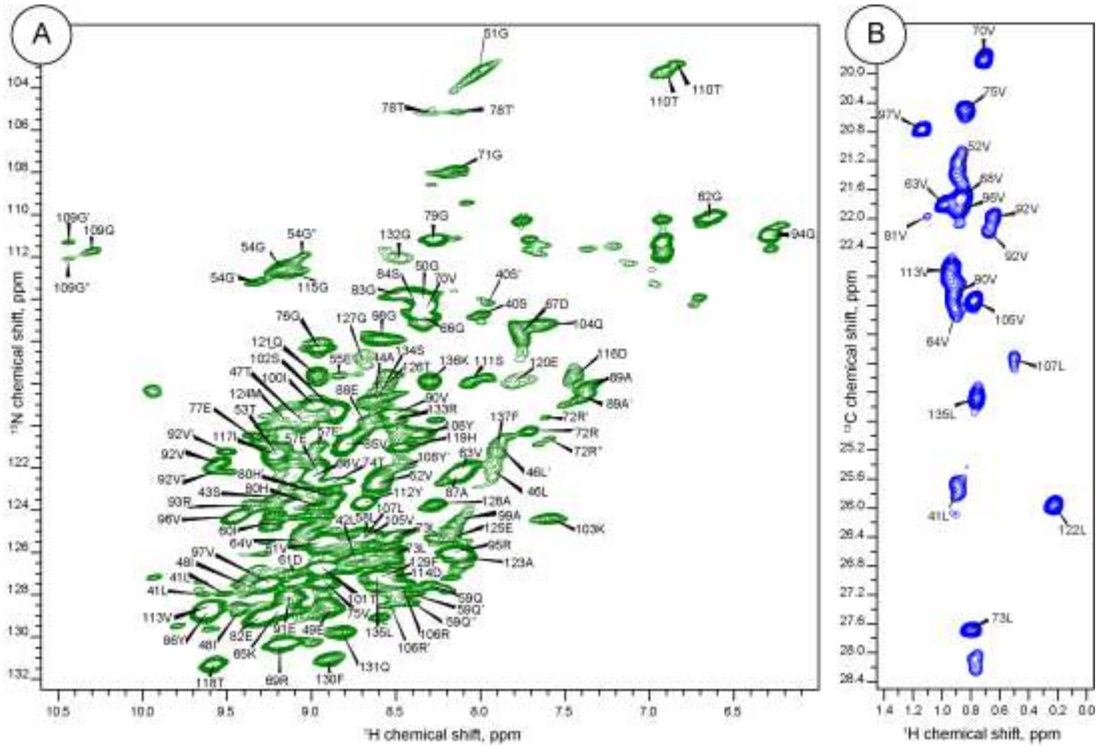

Figure S6. Two-dimensional proton-detected (H)NH and (H)CH correlation spectra of 50% back-protonated [U-^{2}H,^{15}N,^{13}C-Me-pro-S-VL] BacA. Cross-polarization was employed for the inter-spin magnetization transfer. Spectra were obtained on a 900 MHz spectrometer at an MAS rate of 40 kHz. The perdeuterated protein was back protonated at amide sites at a level of 50%. The prime and double prime signs refer to the signals from other polymorphs. Maximum acquisition times in the ^{15}N and ^{13}C dimensions were 40 ms and 29.3 ms, respectively. Prior to Fourier transformation, the data in the proton dimension were truncated to 16 ms and apodised with a 47°-shifted squared sine bell window function. In the carbon and nitrogen dimension, 2 Hz of Lorentzian broadening was applied prior to apodisation. The average nitrogen and carbon linewidths measured without apodisation were 37± 4 Hz and 43± 6 Hz, respectively. The spectra in panels A and B were recorded using four and eight scans, respectively. The inter-scan delay was set to 2.5 s for both spectra.

Figure S6 demonstrates a high spectral resolution, allowing for almost single-residue proton characterization. The available resolution shows that many residues exhibit signal splitting owing to sample polymorphism, as shown in Figure S7.

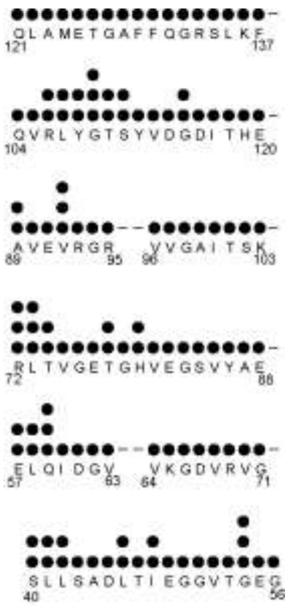

Figure S7. Schematic representation of signal splitting of backbone amide signals. Each circle represents a single signal. Residues are schematically distributed in six vertically aligned windings according to the protein topology.

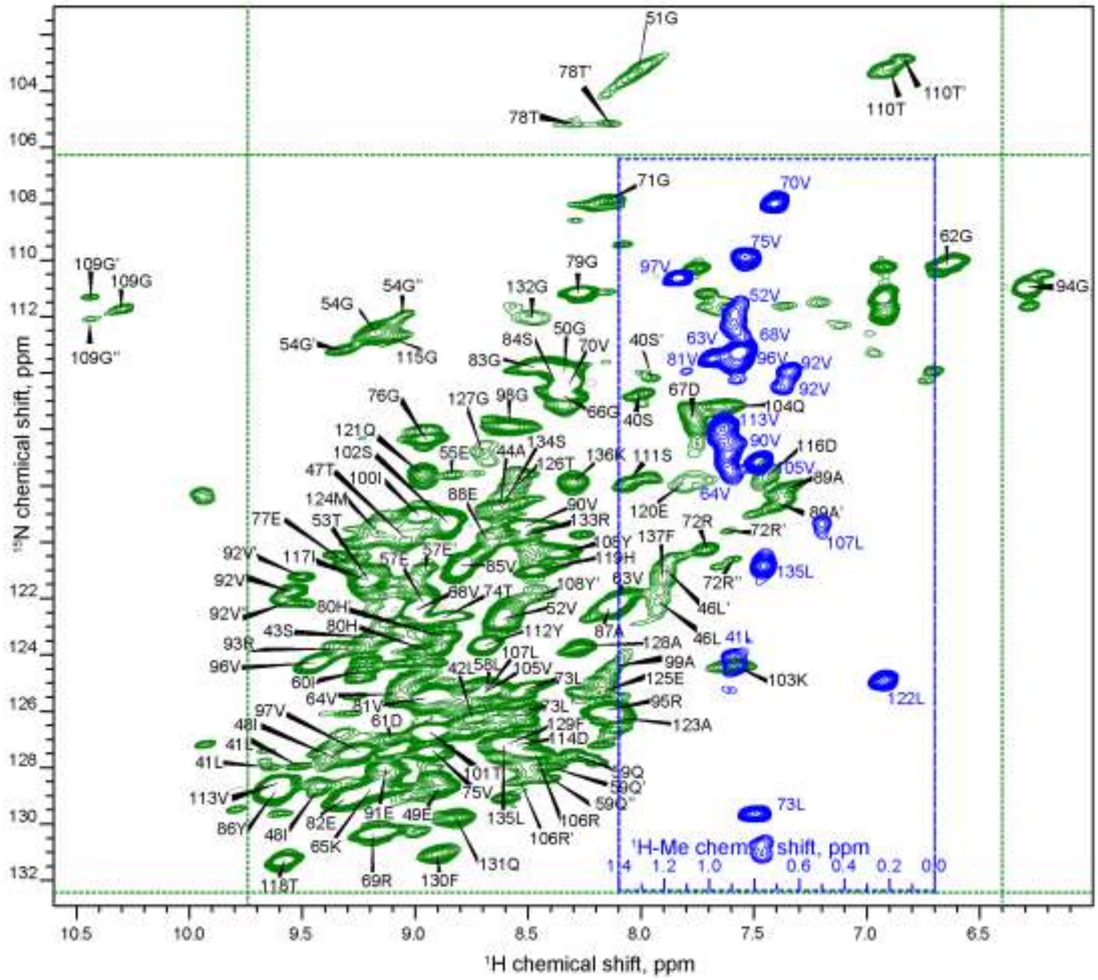

Figure S8. Representation of signal folding in indirect proton (t1) and nitrogen/carbon (t2) dimensions. The amide and methyl resonances are shown in green and blue, respectively. The experimental frequency bandwidth was defined by the time increments in the indirect dimensions. The green dashed lines represent the spectral bandwidths of the amide region and the blue dashed lines represent the aliased methyl regions.

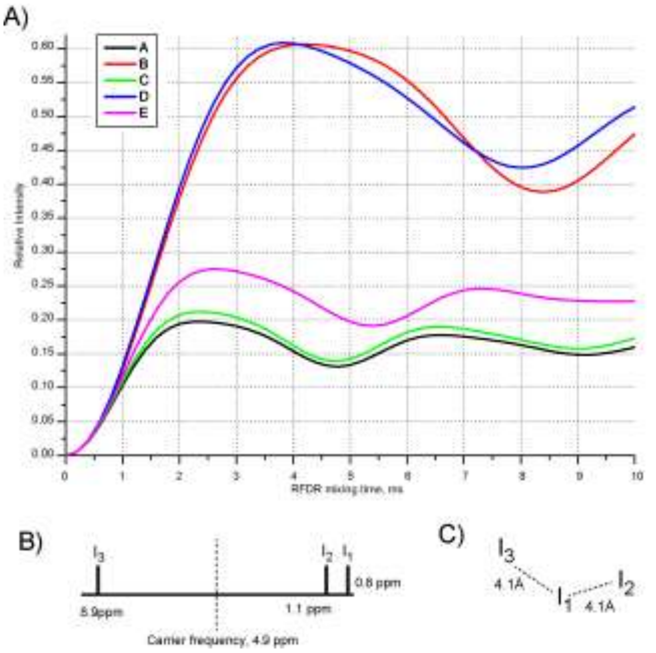

Figure S9. A) Simulated proton-proton magnetization transfer dynamics under fp-RFDR-16 recoupling in a system of two or three interacting protons. The proton RF field strength is 90.9 kHz and the initial magnetizations of I_1, I_2, and I_3 are 1, 0, and 0, respectively. Table S4 summarizes the inter-proton couplings employed in the calculations.

Curve	Dipolar interacting spin pairs	Observed spin	spinsys {<br>dipole 1 2 -1743. 0 0 0<br>dipole 1 3 -1743. 120 0 0<br>dipole 2 3 -1743. 150 0 0<br>shift 1 -4.1p 7.7p 0.65 17 84 12<br>shift 2 -3.8p 7.7p 0.65 77 54 22<br>shift 3 4.0p 7.7p 0.65 20 45 56<br>}
A	I_1 and I_3 ; I_1 and I_2	I_2	
B	I_1 and I_2	I_2	
C	I_1 and I_3 ; I_1 and I_2	I_3	
D	I_1 and I_3	I_3	
E	I_1 and I_3 ; I_1 and I_2 ; I_3 and I_2	I_3	

Table S4. The active proton-proton couplings used for the simulations that are presented in Figure S9. The right column exemplifies the “spin system” section of the SIMPSON input file for the curve “E.” The other simulations used the same parameters. The proton 180° pulse duration was set to 5.5 μs, to match the experimental conditions.

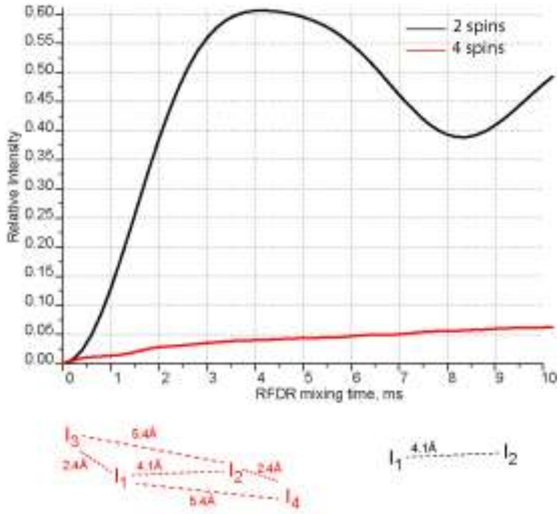

Figure S10. Simulated proton-proton magnetization transfer dynamics under fp-RFDR-16 recoupling in a system of two or four interacting protons. The proton RF field strength was 90.9 kHz and the initial magnetization of I_1 was 1, while the other nuclei had 0 magnetization and I_{2z} was observed. The black curve shows the I_{2z} magnetization dynamics in the system of I_1 and I_2 nuclei, whereas the red curve represents the magnetization in a 4-spin system. Considered proton-proton dipolar couplings are represented by dashed lines along the distances in the bottom figures. The proton systems in the bottom panel are color-coded according to the curves in the main panel. The "spin system" section of the SIMPSON input file is described below. The spin system in red color represents a simplified hydrogen network in a fully protonated system consisting of two adjacent amides and two Hα protons.

```
spinsys {
    dipole 1 2 -1743. 0 0 0
    dipole 1 3 -8689. -45 0 0
    dipole 2 4 -8689. 20 62 0
    dipole 3 2 -763. -11 0 0
    dipole 4 1 -763. 33 18 0

     shift 1 -4.1p 7.7p   0.65  17  84  12
     shift 2 -3.8p 7.7p 0.65  77 54 22
     shift 3 -3.5p 7.7p 0.65   20 45 56
     shift 4 -4.4p 7.7p 0.65   11 15 47
  }
```

References

[1] R. Linser, V. Chevelkov, A. Diehl, B. Reif *J. Magn. Reson.* **2007**, *189*, 209.
[2] U. Akbey, S. Lange, W. Trent Franks, R. Linser, K. Rehbein, A. Diehl, B. J. van Rossum, B. Reif, H. Oschkinat *J. Biomol. NMR*. **2010**, *46*, 67.
[3] L. Thomas, M. Ernst *Magn. Reson.* **2024**, *5*, 153–166.
